# Feeding Preferences of *Agrilus zanthoxylumi* (Coleoptera: Buprestidae) in Relation to Host Plant Volatiles

**DOI:** 10.3390/insects17010088

**Published:** 2026-01-13

**Authors:** Yu Qi, Jiayu Meng, Na Jiang, Xinyu Liu, Yuting Wu, Yanyan Bai, Yingying Zhao, Baozhi Liu, Jiating Yang, Yanan Wang, Shouan Xie

**Affiliations:** 1 Key Laboratory of National Forestry and Grassland Administration on Management of Forest Bio-Disaster, College of Forestry, Northwest A&F University, Yangling 712100, China; qiyu427@nwafu.edu.cn (Y.Q.); jiangna_958@163.com (N.J.); xinyuliu2023@nwafu.edu.cn (X.L.); wyt261888@163.com (Y.W.); baiyanyan@nwafu.edu.cn (Y.B.); zhaoyingying@nwafu.edu.cn (Y.Z.); liubaozhi2024@163.com (B.L.); 15333443128@163.com (J.Y.); wynnnqz@gmail.com (Y.W.); 2 Landscape Architecture & Arts College, Northwest A&F University, Yangling 712100, China; jiayumeng@nwafu.edu.cn

**Keywords:** *Agrilus zanthoxylumi*, *Zanthoxylum bungeanum*, feeding preferences, host selection, plant volatiles

## Abstract

The Chinese pepper buprestid beetle, *Agrilus zanthoxylumi* Li Meng Lou, 1989 (Coleoptera: Buprestidae), is a major trunk-boring pest affecting the yield and quality of *Zanthoxylum bungeanum*. Clarifying its feeding preferences among different pepper varieties and their associations with host-derived volatiles is important for understanding and improving effective management strategies. This study conducted feeding tests under no-choice and dual-choice conditions to evaluate the beetle’s feeding preferences among three *Z. bungeanum* varieties—Fugu, Dahongpao, and Feng. Gas chromatography-mass spectrometry (GC-MS) was employed to analyze leaf volatiles, with factor analysis and partial least squares discriminant analysis (PLS-DA) used to identify key volatiles. Results showed that *A. zanthoxylumi* exhibited the highest resting frequency and feeding amount on *Z. bungeanum* Fugu leaves, with the lowest on *Z. bungeanum* Dahongpao leaves. Significant differences in unique volatiles were observed among the three varieties. *Z. bungeanum* Fugu contained 17 unique components, including (E)-4-hexen-1-ol, (−)-limonene, and (−)-α-pinene, with significantly higher quantities than *Z. bungeanum* Dahongpao and *bungeanum* Feng. Multivariate analyses further revealed distinct distributions in volatiles, with γ-terpinene, α-terpineol, and linalyl acetate emerging as key compounds distinguishing varieties. These results indicate that the feeding preferences of *A. zanthoxylumi* are closely related to host volatiles, suggesting variety-specific compounds may serve as primary chemical cues driving its preferences.

## Simple Summary

Understanding the feeding preferences of *Agrilus zanthoxylumi* Li Meng Lou, 1989 (Coleoptera: Buprestidae) adults across different *Zanthoxylum bungeanum* varieties and their underlying chemical drivers is important for developing sustainable pest management strategies. This study assessed adult feeding preferences on leaves of *Z. bungeanum* Fugu, *Z. bungeanum* Dahongpao, and *Z. bungeanum* Feng varieties and analyzed leaf volatiles to identify key differential compounds. We found that *Z. bungeanum* Fugu leaves were most preferred by adults. The volatile compositions differed markedly among the three varieties, with *Z. bungeanum* Fugu containing the most unique compounds. Factor analysis and partial least squares discriminant analysis (PLS-DA) further identified key differentiating compounds, including (E)-4-hexen-1-ol, (−)-limonene, (−)-α-pinene, γ-terpinene, α-terpineol, and linalyl acetate. The study suggested that host selection by *A. zanthoxylumi* may be driven by key volatiles that exhibit high concentrations and pronounced inter-varietal differences. These results provide a scientific basis for elucidating its chemical location mechanism and developing attractants, repellents, and environmentally friendly pest management strategies.

## 1. Introduction

The genus *Agrilus* was established by Curtis in 1825 and represents one of the most species-rich genera within the family Buprestidae. To date, over 3000 species have been described globally, with more than 280 species recorded in China. All *Agrilus* species are herbivorous, and their extraordinary species diversity is widely considered to be closely associated with the diversity of their host plants [1]. Several *Agrilus* species have been classified as quarantine pests, and some have exhibited strong invasiveness beyond their native ranges, leading to substantial ecological impacts and economic losses [2]. *Agrilus zanthoxylumi* Li Meng Lou, 1989 (Coleoptera: Buprestidae) is a destructive trunk-boring pest of *Zanthoxylum bungeanum*, noted for its cryptic behavior, prolonged damage cycle, and high mortality of infested trees [3,4]. *A. zanthoxylumi* adults feed on *Z. bungeanum* leaves to replenish nutrients and oviposit on bark scars or cracks [5,6]. After hatching, larvae bore into the phloem and gradually invade the cambium, forming slender, meandering feeding tunnels [4,7,8]. Severe infestations have been shown to disrupt nutrient transport within the tree, resulting in gummosis, necrosis, desiccation, and fissuring of the bark. In major *Z. bungeanum*–producing regions, such as Hancheng, Shaanxi Province, infestation rates of *A. zanthoxylumi* in young trees (≤4–5 years old) have been reported to exceed 70%. In affected plantations, damaged trees have been found to contain an average of 28.8 larval galleries, with up to 72 galleries per tree in severely infested stands. Such extensive larval boring has been associated with disruption of vascular tissues, ultimately leading to tree decline or death and annual yield reductions of 25–35%, resulting in substantial economic losses to the local pepper industry [9,10,11,12]. At present, *Z. bungeanum* cultivation areas still rely on chemical pesticides to control this pest, but the long-term use of a single pesticide is prone to causing the ‘3R’ problem—resistance, resurgence, and residue [8]. Consequently, developing environmentally friendly and effective control strategies has become an urgent priority in *A. zanthoxylumi* management.

Through long-term coevolution, herbivorous insects and their host plants have established complex chemical communication systems, in which host-released volatile organic compounds (VOCs) play pivotal roles in mediating insect behavior [13,14,15]. The host selection process for herbivorous insects typically involves searching, identifying, and feeding. Plant volatiles not only attract insects over long distances through olfactory cues and guide their positioning, but also influence their assessment of host quality and feeding behavior once they have landed [16,17]. These VOCs primarily consist of alcohols, aldehydes, ketones, esters, olefins, and terpenoids [18,19,20]. Specific compounds or their combinations may enhance host attractiveness, promote insect feeding or oviposition, whereas others exert deterrent or repellent effects [21,22,23]. For example, host-specific leaf volatiles, including (Z)-3-hexenol, hexyl acetate, (E)-β-ocimene, and linalool, have been shown to play important roles in host plant location in adult *Agrilus mali* [24].

The composition and relative contents of volatiles differ between plant species and even between varieties of the same plant. This chemical differentiation is a key factor underlying variation in the feeding preferences of herbivorous insects [24,25,26]. Research indicates that the characteristic host volatiles released by different varieties of the same plant serve as chemical cues for insect host selection, directly influencing their feeding preferences and fitness [27]. For example, *Agrilus planipennis* exhibited clear differences in leaf selection and feeding behavior among different *Fraxinus* species. Adults preferentially feed on *F. pennsylvanica*, *F. americana*, and *F. nigra*, whereas significantly lower preference was observed for *F. mandshurica*, *F. quadrangulata*, and *F. excelsior*. Comparative analyses indicated that antennally active leaf volatiles differ in both composition and relative abundance among these hosts, and that preferred hosts tend to emit relatively lower total amounts of volatiles [26]. Similarly, *Ceuthorrhynchus asper* exhibited marked feeding preferences among rapeseed varieties. The preferred varieties CKE58 and Qingza 5 contained higher levels of volatiles such as (Z)-3-hexenyl acetate, hexyl acetate, and trans-3-hexen-1-ol, which might have played a pivotal role in host selection of these insects [20].

Characteristic volatiles released by host plants not only mediate insect host selection and feeding preferences, but also hold great potential for pest trapping, population monitoring, and environmentally sustainable pest control [23,28]. Previous studies by our research group have indicated that *A. zanthoxylumi* adults exhibit host specialization and cause differential levels of damage among different *Z. bungeanum* varieties [6]. However, the relationship between the feeding preferences of *A. zanthoxylumi* and the volatile profiles of its *Z. bungeanum* hosts remains largely unexplored. Therefore, this study employed *Z. bungeanum* Fugu, *Z. bungeanum* Dahongpao, and *Z. bungeanum* Feng as test plants, integrating feeding preference experiments with volatile analyses to systematically compare the feeding preferences of *A. zanthoxylumi* adults among different host varieties and to identify key volatile compounds potentially associated with host selection and feeding behavior. Specifically, this study aimed to determine volatiles closely related to the feeding behavior of *A. zanthoxylumi*, thereby providing scientific evidence for elucidating its host selection mechanisms and for the development of novel, environmentally friendly control methods.

## 2. Materials and Methods

### 2.1. Insect Collection

*Z. bungeanum* branches containing overwintering larvae of *A. zanthoxylumi* were collected in April 2023 from a pepper orchard in Xi Lijiagou Village, Lantian County, Xi’an City, Shaanxi Province, China (34°15′ N, 109°45′ E). To prevent desiccation, the cut ends of the branches were sealed with plastic film before being transported to the laboratory. The branches were placed in an artificial climate chamber maintained at a temperature of 26 ± 1 °C, relative humidity of 60 ± 5%, and a 16:8 h light–dark photoperiod. Adult eclosion was monitored daily, with newly emerged individuals placed into rearing cages according to their eclosion age. Fresh leaves from all three *Z. bungeanum* varieties used in the experiments and moistened cotton balls were provided to supplement nutrition and hydration for the adults.

### 2.2. Plant Collection

The test varieties included *Z. bungeanum* Fugu, *Z. bungeanum* Dahongpao, and *Z. bungeanum* Feng, all collected from the same orchard in Xi Lijiagou Village, Lantian County. For each variety, leaves were collected from three independent and healthy trees, which were treated as biological replicates. From each tree, leaves were randomly sampled from five branches and pooled to form one composite sample per tree for subsequent volatiles extraction.

### 2.3. Host Selection of A. zanthoxylumi Adults

To assess the host selection behavior of *A. zanthoxylumi* adults among different *Z. bungeanum* varieties, choice experiments were conducted in a cylindrical plastic container (40 cm diameter, 25 cm height) lined with approximately 5 cm of moistened sand at the bottom. Fresh branches bearing eight leaves each of *Z. bungeanum* Fugu, *Z. bungeanum* Dahongpao, and *Z. bungeanum* Feng were inserted equidistantly from three directions. Prior to the commencement of the experiments, twelve adult insects within one week of eclosion were released into the box; both males and females were used without sex selection to evaluate the overall host selection response. The box was covered with 120-mesh netting at the top. The experiments were conducted under controlled environmental conditions of 26 ± 1 °C, 60 ± 5% relative humidity, and a 16:8 h light–dark photoperiod. Observations were conducted from 08:00 to 20:00 daily, and the number of adults resting on leaves of each variety was recorded every two hours. At each observation time, only adults that rested and remained on the leaves of each *Z. bungeanum* variety were counted. Each experiment was replicated six times.

### 2.4. Feeding Preferences of A. zanthoxylumi on Different Z. bungeanum Varieties

Adult feeding experiments were conducted using the leaf-disc method [24]. The leaf mass (*Y*) and leaf area (*X*) of the three varieties were first measured using a leaf area meter (Regent Instruments Inc., Quebec, QC, Canada), and a linear regression model (*Y* = *aX* + *b*) was established to calculate the feeding mass. Subsequently, standard leaf discs were prepared using a 2.5 cm diameter leaf punch and placed in glass Petri dishes lined with moist filter paper to maintain disc moisture. Adults (sex-unselected) within one week after eclosion were starved for 24 h before experiments. One *A. zanthoxylumi* per dish was allowed to feed for 48 h. After feeding, the remaining leaf area was measured, and the consumed area was converted to feeding mass using the established linear regression model.

Two experimental conditions were tested: (i) No-choice condition—each Petri dish contained leaf discs from only one variety; (ii) Dual-choice condition—three combinations (*Z. bungeanum* Fugu × *Z. bungeanum* Dahongpao, *Z. bungeanum* Fugu × *Z. bungeanum* Feng, *Z. bungeanum* Dahongpao × *Z. bungeanum* Feng) were established, with two discs placed approximately 3 cm apart in each dish. To avoid potential positional bias, the relative positions of the two leaf discs were alternated among replicates. Each experiment was replicated six times under identical laboratory conditions as described in Section 2.3 (26 ± 1 °C, 60 ± 5% relative humidity, and a 16:8 h light–dark photoperiod).

### 2.5. Extraction and Analyses of Z. bungeanum Volatiles

Approximately 5 g of healthy leaves from each biological replicate of *Z. bungeanum* were cut into small pieces, ground into a homogenate, and transferred into a 20 mL sample vial, occupying approximately 45% of the sample vial. The sample vial was sealed and equilibrated at 50 °C for 10 min to allow full saturation. Volatiles were extracted using solid-phase microextraction (SPME) with a 50/30 μm divinylbenzene/carboxen/polydimethylsiloxane (DVB/CAR/PDMS) fiber (gray color). This fiber is suitable for the adsorption of a broad range of volatile compounds, particularly green leaf volatiles. The extraction was performed for 30 min, after which the fiber was thermally desorbed in the GC–MS injector at 250 °C for 3 min.

GC–MS was performed using an ISQ single quadrupole GC–MS system (Thermo Fisher Scientific, Waltham, MA, USA) equipped with a DB-5ms capillary column (30 m × 0.25 mm × 0.25 μm). Helium (purity 99.999%) was used as the carrier gas at a constant flow rate of 1.0 mL/min. The injection volume was 10 μL with a split ratio of 10:1, and the injector temperature was maintained at 250 °C. The column temperature program was set as follows: the initial temperature was 60 °C, held for 2.5 min, ramped at 6 °C/min to 190 °C, followed by a 10 °C/min increase to 230 °C, and held for 10 min. Mass spectra were obtained using electron impact ionization (EI, 70 eV) over a scan range of 35–400 amu. Compound identification was performed by comparing mass spectra with those in the NIST2011 library, and relative quantification was achieved based on peak area normalization.

### 2.6. Data Analyses

Differences in host selection, feeding amount under no-choice conditions, and relative contents of volatiles among varieties were analyzed using one-way analysis of variance (ANOVA). Feeding amount under dual-choice conditions was compared using Mann–Whitney *U* test. Factor analysis was conducted on the volatiles of the leaves, with principal varieties extracted by Kaiser normalization and rotated using the Varimax method to optimize the factor loading matrix. Factor scores for each variety were calculated based on the rotated factor loading matrix [24]. Subsequently, factor scores were subjected to one-way ANOVA, and multiple comparisons were performed using Tukey’s HSD test at a significance level of α = 0.05. A two-dimensional score plot was generated based on the scores of composite factors 1 and 2, followed by PLS-DA, which was conducted using the MetaboAnalyst platform [20,29]. All statistical analyses and visualizations were performed using SPSS 26.0 and Microsoft Excel 2016.

## 3. Results

### 3.1. Host Selection of A. zanthoxylumi Adults Among Z. bungeanum Varieties

**Figure 1:**
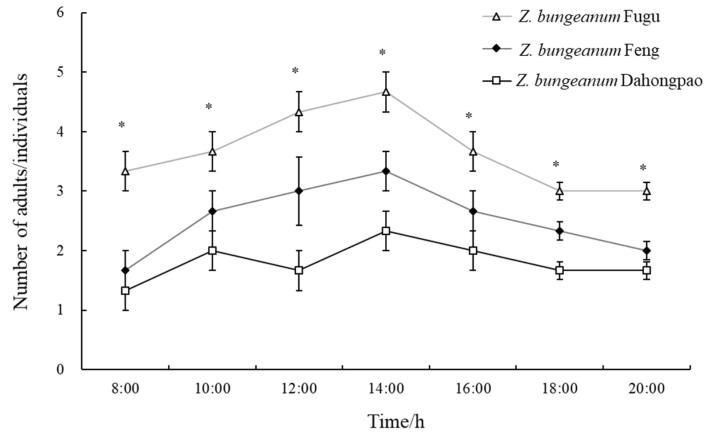
Number of *Agrilus zanthoxylumi* adults (mean ± SE) observed on three *Zanthoxylum bungeanum* varieties at different time points. Asterisks (*) indicate significant differences between *Z. bungeanum* Fugu and the other two varieties (*Z. bungeanum* Feng and *Z. bungeanum* Dahongpao) at the same time point (one-way ANOVA, *p* < 0.05).

During the observation period from 08:00 to 20:00, the number of *A. zanthoxylumi* adults resting on leaves differed significantly among the three *Z. bungeanum* varieties (Figure 1). The number of adults resting on *Z. bungeanum* Fugu leaves was the highest and was significantly higher than that on *Z. bungeanum* Feng and *Z. bungeanum* Dahongpao leaves (*p* < 0.05), while the fewest adults were observed on *Z. bungeanum* Dahongpao leaves. Throughout the observation period, the number of adults resting on both *Z. bungeanum* Fugu and *Z. bungeanum* Feng leaves exhibited a similar pattern, first increasing and then decreasing, with the highest activity observed at 14:00. These results indicated that *A. zanthoxylumi* adults showed a clear preference for *Z. bungeanum* Fugu leaves over the other two varieties.

### 3.2. Feeding of A. zanthoxylumi on Z. bungeanum Leaves

#### 3.2.1. Relationship Between Leaf Area and Mass

**Figure 2:**
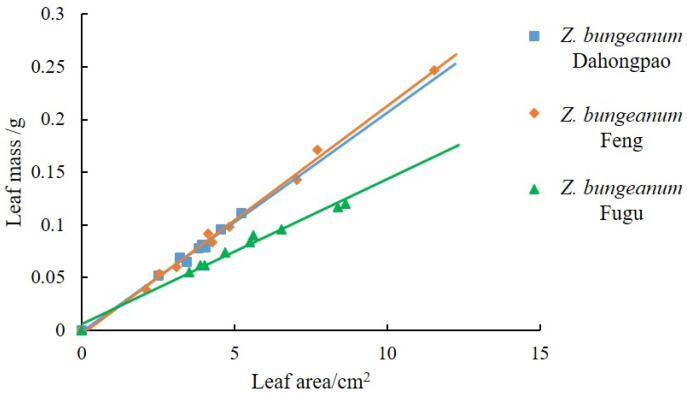
Linear relationship between leaf area and leaf mass for three *Z. bungeanum* varieties. Each point represents an individual leaf measurement, with *Z. bungeanum* Dahongpao (blue squares), *Z. bungeanum* Feng (orange diamonds), and *Z. bungeanum* Fugu (green triangles). Separate linear regression lines are fitted for each variety in the corresponding colors.

A significant positive linear relationship was observed between leaf area and leaf mass for all three *Z. bungeanum* varieties (Figure 2). The regression equations were as follows: *Z. bungeanum* Dahongpao: *Y* = 0.0208*X* − 0.0012 (*R*^2^ = 0.9895); *Z. bungeanum* Feng: *Y* = 0.0217*X* − 0.0036 (*R*^2^ = 0.9956); *Z. bungeanum* Fugu: *Y* = 0.0137*X* + 0.0063 (*R*^2^ = 0.9868). These results demonstrated that as leaf area increases, leaf mass also increases steadily. The relationship between leaf area and mass varies among different varieties, with *Z. bungeanum* Fugu leaves exhibiting the smallest increase in mass.

#### 3.2.2. Feeding Under No-Choice Conditions

**Figure 3:**
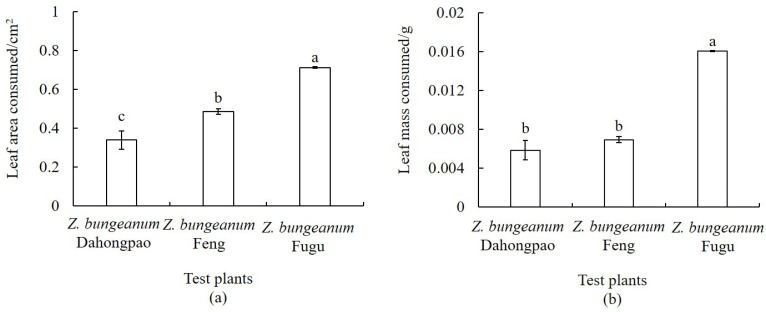
Feeding performance by *A. zanthoxylumi* adults (mean ± SE) on three *Z. bungeanum* varieties under no-choice conditions. (**a**) Leaf area consumed by adults on three *Z. bungeanum* varieties. (**b**) Leaf mass consumed by adults on three *Z. bungeanum* varieties. Different lowercase letters indicate significant differences in the insects’ feeding among varieties (one-way ANOVA, *p* < 0.05).

Under no-choice conditions, the feeding area of *A. zanthoxylumi* adults differed significantly among the three *Z. bungeanum* varieties—*Z. bungeanum* Dahongpao, *Z. bungeanum* Feng, and *Z. bungeanum* Fugu (Figure 3a, *p* < 0.05). Adults consumed the largest leaf area of *Z. bungeanum* Fugu (0.7127 ± 0.0031 cm^2^), which was significantly higher than that of *Z. bungeanum* Feng (0.4857 ± 0.0145 cm^2^) and *Z. bungeanum* Dahongpao (0.3393 ± 0.0473 cm^2^). Based on the linear regression relationship between leaf area and leaf mass (Figure 3b), the corresponding feeding mass was calculated. The results showed that *Z. bungeanum* Fugu leaves also exhibited the highest feeding mass (0.0161 ± 0.0001 g), significantly exceeding that of *Z. bungeanum* Feng and *Z. bungeanum* Dahongpao, while no significant difference was observed between *Z. bungeanum* Feng (0.0069 ± 0.0003 g) and *Z. bungeanum* Dahongpao (0.0059 ± 0.0010 g) (*p* > 0.05).

#### 3.2.3. Feeding Under Dual-Choice Conditions

Under dual-choice conditions, significant differences in both feeding area and feeding mass were observed between the two host varieties within each pairwise combination (*p* < 0.05). As shown in Table 1, in terms of feeding area, adults exhibited a significantly larger feeding area on *Z. bungeanum* Fugu than on *Z. bungeanum* Feng and *Z. bungeanum* Dahongpao, while the feeding area on *Z. bungeanum* Feng (2.64 ± 0.11 cm^2^) was also significantly greater than that on *Z. bungeanum* Dahongpao (1.50 ± 0.22 cm^2^). Regarding feeding mass, adults showed a consistent pattern of selectivity. The feeding mass on *Z. bungeanum* Fugu leaves was significantly higher than that on *Z. bungeanum* Feng (0.037 ± 0.003 g) and *Z. bungeanum* Dahongpao (0.023 ± 0.003 g). Within the *Z. bungeanum* Feng × *Z. bungeanum* Dahongpao combination, adults consumed significantly more leaf mass from *Z. bungeanum* Feng (0.054 ± 0.002 g) than from *Z. bungeanum* Dahongpao (0.030 ± 0.005 g).

### 3.3. Composition and Relative Contents of Volatiles in Z. bungeanum Leaves

A total of 39 major volatile compounds were identified from the leaves of the three *Z. bungeanum* varieties (Table 2), including alcohols, alkenes, aldehydes, alkanes, esters, ketones, and naphthalenes. Six volatiles were detected in all three varieties, namely linalool, sabinene, myrcene, β-caryophyllene, (−)-β-pinene, and γ-terpinene, and their relative contents differed significantly (*p* < 0.05). Among them, *Z. bungeanum* Dahongpao exhibited significantly higher relative contents of linalool (20.11%), sabinene (8.89%), and γ-terpinene (0.65%) than *Z. bungeanum* Feng and *Z. bungeanum* Fugu. The relative content of myrcene in *Z. bungeanum* Feng (6.91%) was significantly greater than that in *Z. bungeanum* Fugu, whereas β-caryophyllene was most abundant in *Z. bungeanum* Fugu, significantly exceeding that in *Z. bungeanum* Dahongpao and *Z. bungeanum* Feng.

Moreover, distinctive constituents were present in the volatiles of all three *Z. bungeanum* varieties. The unique compounds detected in *Z. bungeanum* Dahongpao included linalyl acetate, (+)-α-pinene, trans-3-hexen-1-ol, α-terpineol and bicyclo[3.1.0]hex-2-ene. *Z. bungeanum* Fugu exhibited seventeen unique compounds, such as (−)-α-pinene, γ-terpinene and (E)-4-hexen-1-ol. *Z. bungeanum* Feng contained only one unique compound, germacrene D. Although the three varieties shared a certain degree of similarity in the major volatile components, they differed markedly in their relative contents and the presence of variety-specific compounds.

### 3.4. Factor Analysis of Volatiles in Z. bungeanum Leaves

To characterize the volatile compounds among different *Z. bungeanum* varieties, factor analysis was performed on the leaf volatile compounds of *Z. bungeanum* Fugu, *Z. bungeanum* Dahongpao, and *Z. bungeanum* Feng. Two factors were extracted, accounting for 94.40% of the total variance (Table 3), indicating that the two factors effectively captured the dominant information in the volatile dataset. Factor 1 explained 66.53% of the total variance, with twenty-eight compounds exhibiting strong contributions (absolute loadings > 0.7). Among these, (−)-limonene, (−)-α-pinene, α-muurolene, (−)-α-cubebene, and (+)-aromadendrene showed the highest loading values, at 0.961, 0.960, 0.960, 0.960, and 0.960, respectively. The lowest loading values were observed for terpinyl acetate, (+)-dipentene, myrcene, (1R)-(+)-α-pinene, and cineole, with values of −0.969, −0.968, −0.965, −0.884, and −0.872, respectively. Factor 2 explained 27.87% of the total variance and included nine major contributing volatiles (absolute loadings > 0.7). Bicyclo[3.1.0]hex-2-ene, linalyl acetate, sabinene, α-terpineol, and (+)-α-pinene exhibited the highest loading values, at 0.968, 0.958, 0.956, 0.955, and 0.953, respectively. In contrast, α-humulene and germacrene D showed the lowest loading values, measured at −0.699 and −0.682, respectively. The volatile components constituting each factor exhibited significant score differences across the three varieties. Specifically, within Factor 1 and Factor 2, the volatile components with the highest contributions originated from *Z. bungeanum* Fugu and *Z. bungeanum* Dahongpao, respectively.

**Figure 4:**
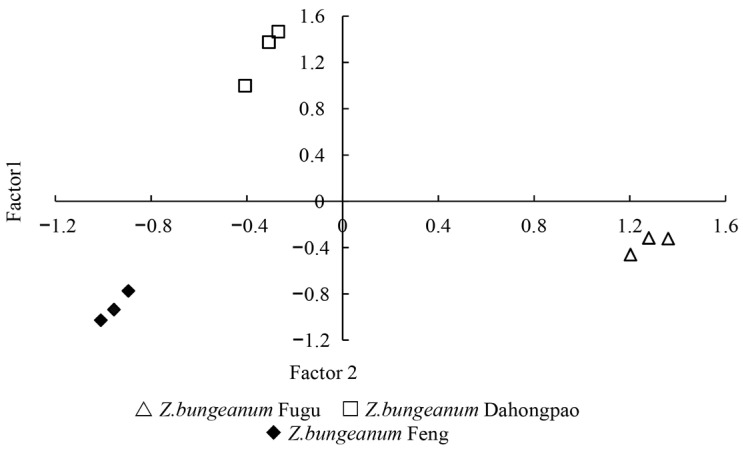
Factor analysis score plot illustrating the distribution patterns of leaf volatiles among the three *Z. bungeanum* varieties. Triangles indicate *Z. bungeanum* Fugu, squares indicate *Z. bungeanum* Dahongpao, and diamonds indicate *Z. bungeanum* Feng. Factor 1 and Factor 2 together explain the major variance in volatile compositions.

A two-dimensional scatter plot based on the scores of Factor 1 and Factor 2 (Figure 4) revealed a clear differentiation in the volatile compounds among the three *Z. bungeanum* varieties. *Z. bungeanum* Fugu clustered in the fourth quadrant and exhibited the highest scores on Factor 1 (1.28 ± 0.08 a), indicating that its characteristic volatiles were predominantly associated with the components represented by this factor. *Z. bungeanum* Dahongpao was distributed in the second quadrant and showed the highest scores on Factor 2 (1.28 ± 0.25 a). In contrast, *Z. bungeanum* Feng was positioned in the third quadrant, displaying negative scores on both Factor 1 and Factor 2 (−0.95 ± 0.06 c, −0.91 ± 0.13 b), suggesting relatively weak associations with the volatile components represented by either factor. Overall, the volatile compounds of *Z. bungeanum* Fugu differed markedly from those of the other two varieties, reflecting its distinct chemical composition.

### 3.5. PLS-DA of Volatiles in Z. bungeanum Leaves

**Figure 5:**
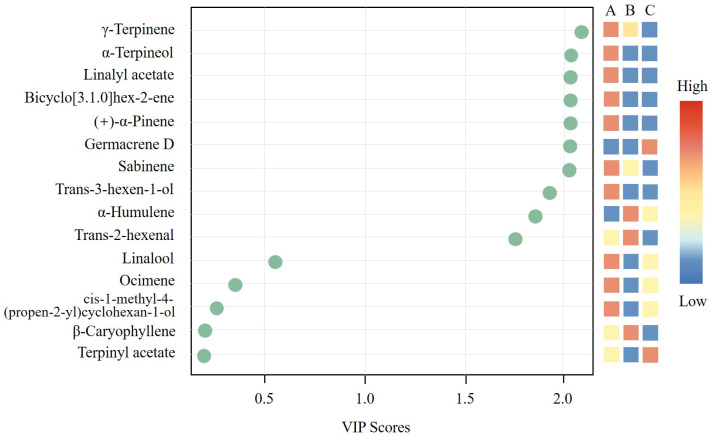
Comparison of the top 15 volatiles with the highest VIP scores identified by partial least squares discriminant analysis (PLS-DA) among the three *Z. bungeanum* varieties. A represents *Z. bungeanum* Dahongpao; B represents *Z. bungeanum* Fugu; C represents *Z. bungeanum* Feng. Green circles indicate the VIP scores of individual volatile compounds, with higher values reflecting greater contributions to the discrimination among varieties. The colored blocks on the right represent the relative contents of each compound across the three varieties.

To further verify the differences in volatile organic compounds among the three *Z. bungeanum* varieties, PLS-DA was conducted (Figure 5). The model clearly separated the three *Z. bungeanum* varieties, indicating significant differences in the volatile compound composition among the varieties. Based on the variable importance in projection (VIP) scores, ten compounds with VIP values greater than 1.0 were identified as key contributors to varietal discrimination. Among these, γ-terpinene, α-terpineol, linalyl acetate, bicyclo[3.1.0]hex-2-ene, and (+)-α-pinene exhibited the highest VIP values and thus represent the major chemical markers distinguishing the three *Z. bungeanum* varieties.

## 4. Discussion

The role of plant VOCs in mediating host selection and feeding preferences of insects has been widely documented in multiple *Agrilus* species. For instance, adults of *A*. *mali* exhibited distinct preferences among four Rosaceae host species, showing the highest frequency of selection and feeding on *Malus halliana* [24]. Variations in the proportion of host-specific volatiles have been proposed as a key factor driving its selective feeding behavior. Similarly, *A. planipennis* adults demonstrated pronounced feeding preferences among different *Fraxinus* varieties, with resistant *Fraxinus mandshurica* leaves containing higher concentrations of 11 VOCs compared to the susceptible *Fraxinus pennsylvanica* [26]. Collectively, these results underscore the crucial role of host plant volatiles in governing insect host selection and feeding preferences. Further studies have demonstrated that plant volatiles exert a key regulatory influence on host recognition, orientation, and feeding behaviors across a wide range of phytophagous insects [13,23,27].

Therefore, investigating the relationship between the feeding preferences of *A. zanthoxylumi* and host-plant VOCs is important for identifying key semiochemicals involved in host discrimination and for developing effective plant-derived attractants. In this study, *A. zanthoxylumi* adults exhibited a clear feeding preference among the three *Z. bungeanum* varieties, with significantly higher selection frequency and feeding amount on *Z. bungeanum* Fugu than on *Z. bungeanum* Feng or *Z. bungeanum* Dahongpao. This result is consistent with previous observations and suggests that differences in the volatile compositions among *Z. bungeanum* varieties may play an important role in shaping host preferences [6].

To identify the volatiles potentially responsible for this differential host selection, leaf VOCs from the three *Z. bungeanum* varieties were extracted and analyzed under the experimental conditions used in this study. A total of 39 major volatiles were identified, including six compounds shared among all varieties but differing significantly in their relative contents. Each variety also exhibited distinct volatile profiles, with *Z. bungeanum* Fugu containing 17 unique compounds, such as (−)-α-pinene, (E)-4-hexen-1-ol, and (−)-limonene; *Z. bungeanum* Dahongpao containing five unique volatiles, including (+)-α-pinene, linalyl acetate, and α-terpineol; and *Z. bungeanum* Feng producing only one exclusive component, germacrene D. These results indicate pronounced inter-varietal differences in leaf volatile composition and relative abundance. It should be noted that the present study was designed to compare volatile profiles among varieties and did not aim to distinguish between constitutively emitted compounds and those potentially induced by biotic stimuli. Further studies incorporating controlled herbivore-induced or simulated damage treatments would be valuable for clarifying the inducibility and ecological roles of these volatile compounds.

Previous studies have demonstrated that (E)-4-hexen-1-ol significantly enhances the olfactory response of *P. xylostella* to host plant odors, either alone or in combination with other green-leaf volatiles [30]. Low concentrations of (−)-limonene exerted a strong attractant effect on *Nilaparvata lugens*, increasing field trap catches by 1.8-fold within 24 h. Furthermore, (−)-α-pinene has been shown to attract several species of bark beetles, whereas the presence of (+)-α-pinene reduces its trapping efficiency [31,32]. Taken together, we hypothesize that the unique volatile components identified in *Z. bungeanum* Fugu leaves may play a key attractive role in the host selection and feeding processes of *A. zanthoxylumi*, thereby resulting in the stronger feeding preference observed for this variety.

Factor analysis further confirmed the systematic differences in volatile compositions among the three *Z. bungeanum* varieties. As a widely used multivariate statistical method, factor analysis can reveal the structural characteristics of complex volatile datasets while retaining the major information, and the construction of composite factors allows for the identification of potential associations among compounds [33,34]. In this study, the two-dimensional scatter plot indicated clear differentiation of the three varieties within the composite factor space, reflecting inherent differences in their overall volatile compounds.

*Z. bungeanum* Fugu samples clustered in the fourth quadrant and exhibited the highest scores on Factor 1. Factor 1 comprised multiple monoterpenes and sesquiterpenes, which play critical roles in insect host-location and recognition; their overall content and combination patterns often determine the strength of host-plant chemical cues perceived by insects [35,36]. The high loading of *Z. bungeanum* Fugu on Factor 1 suggests that its volatile composition aligns closely with this principal component, potentially forming a chemical background that is more easily recognized by *A. zanthoxylumi*, consistent with the stronger feeding preference observed. In contrast, *Z. bungeanum* Dahongpao exhibited the highest scores on Factor 2, indicating that its volatile composition is more strongly influenced by the chemical dimension represented by this factor. Although Factor 2 explained a smaller proportion of the total variance than Factor 1, it encompassed several volatiles known to play ecological roles in plant–insect interactions [37,38], suggesting that host recognition in *Z. bungeanum* Dahongpao may depend on a more specific combination of chemical cues.

PLS-DA analysis can identify key chemical constituents responsible for differentiating varieties under conditions of multivariate correlation, thereby effectively screening volatiles that contribute significantly to varietal discrimination [20,29]. In this study, among the key volatiles with VIP scores greater than 1.0, γ-terpinene, α-terpineol, and linalyl acetate played particularly important roles in distinguishing the three *Z. bungeanum* varieties. Notably, γ-terpinene exhibited the highest VIP value. As a common volatile across all three varieties, γ-terpinene was most abundant in *Z. bungeanum* Dahongpao, moderately present in *Z. bungeanum* Fugu, and least abundant in *Z. bungeanum* Feng. Combined with the feeding experiment results, it is suggested that γ-terpinene may enhance the feeding preferences of *A. zanthoxylumi* at moderate concentrations, whereas excessively high or low concentrations may reduce its attractiveness. Previous studies have shown that γ-terpinene can elicit significant antennal responses in *Dendrothrips minowai* and *Plutella xylostella* during electroantennogram (EAG) recordings; however, it does not necessarily act as an attractant in behavioral experiments, indicating that electrophysiological responses do not always correlate with behavioral outcomes and that its effect may depend on concentration or compound ratios [39,40].

Furthermore, α-terpineol has been reported to act synergistically with other host-plant volatiles, enhancing the orientation response of *Optatus palmaris* adults [41]. In *Bombus terrestris*, the response to α-terpineol exhibited a typical concentration-dependent effect, with attraction observed at low to moderate concentrations and repulsion at high concentrations [42]. The activity of linalyl acetate is also influenced by the odor background; it can elicit feeding preference in *Antheraea assamensis* larvae and has been identified as a key compound associated with orientation behavior in *Vespa velutina*, with its attractiveness depending on the composition and ratio of background plant volatiles [43,44]. Collectively, these results indicate that these key compounds do not function solely as repellents or attractants, but instead act as modulators of host-recognition cues, with their effects depending on release concentration, component ratios, and the context of co-occurring volatiles [14,15,17,45,46,47].

Overall, the feeding preferences of *A. zanthoxylumi* for different *Z. bungeanum* varieties were closely linked to the emission of characteristic leaf volatiles. This study established a clear relationship between host plant chemical cues and the feeding choice of *A. zanthoxylumi*, providing a theoretical basis for the application of host-derived volatiles in pest monitoring and behavioral regulation. However, the present work focused solely on compositional differences in leaf volatiles and did not experimentally validate the behavioral or electrophysiological activity of individual compounds. Future research should integrate EAG, gas chromatography–electroantennographic detection (GC-EAD), and behavioral experiments to elucidate the functional roles of key VOCs identified in this study, and further combine molecular approaches to validate the involvement of olfactory-related genes in mediating host recognition and feeding preference of *A. zanthoxylumi*. Furthermore, optimization of compound mixtures and ratios may facilitate the identification of efficient and stable plant-derived attractants or repellents, contributing to the development of environmentally sustainable control strategies for *A. zanthoxylumi*.

## 5. Conclusions

Our study demonstrated that *A. zanthoxylumi* adults exhibited pronounced feeding preferences among the three *Z. bungeanum* varieties, with both host-selection frequency and feeding amount being highest on *Z. bungeanum* Fugu. Although six volatile compounds were shared across all varieties, their relative abundances differed significantly, and each variety possessed unique components, among which *Z. bungeanum* Fugu contained as many as 17 exclusive volatiles. Both factor analysis and PLS-DA revealed clear differentiation in the volatiles of the three varieties. Integrating the behavioral data with the chemical analyses, we inferred that host selection in *A. zanthoxylumi* may be driven by key volatiles characterized by high abundance and pronounced inter-varietal differences, such as (E)-4-hexen-1-ol, (−)-limonene, (−)-α-pinene, γ-terpinene, α-terpineol, and linalyl acetate. Further research is required to elucidate the specific functions of these host-related volatiles, thereby enabling the development of environmentally friendly control strategies targeting *A. zanthoxylumi*. This will provide a scientific basis for the sustainable management of *Z. bungeanum* plantations.

## Figures and Tables

**Table 1:** Feeding performance of *A. zanthoxylumi* adults (mean ± SE) on three *Z. bungeanum* varieties under dual-choice conditions. For each variety pair, asterisks (*) indicate significant differences in feeding area or feeding mass between the two varieties (Mann–Whitney U test, *p* < 0.05).

*Z. bungeanum* Variety Pair	*Z. bungeanum* Variety	Feeding Area (cm^2^)	Feeding Mass (g)
Fugu × Feng	Fugu	3.65 ± 0.23 *	0.056 ± 0.003 *
	Feng	1.89 ± 0.12	0.037 ± 0.003
Fugu × Dahongpao	Fugu	3.26 ± 0.82 *	0.051 ± 0.011 *
	Dahongpao	1.15 ± 0.12	0.023 ± 0.003
Feng × Dahongpao	Feng	2.64 ± 0.11 *	0.054 ± 0.002 *
	Dahongpao	1.50 ± 0.22	0.030 ± 0.005

**Table 2:** Comparison of volatiles and relative contents (mean ± SD) in leaves of three varieties in *Z. bungeanum*. “—” indicates that the compound was detected but its relative content was below 0.5%, while “——” indicates that the compound was not detected. Different lowercase letters in the same row indicate significant differences in the relative content of the same compound among different varieties of *Z. bungeanum* (one-way ANOVA, *p* < 0.05).

Compound Name	Relative Content %		
	*Z. bungeanum* Dahongpao	*Z. bungeanum* Feng	*Z. bungeanum* Fugu
Linalool	20.11 ± 1.29 a	9.76 ± 2.07 b	0.96 ± 0.06 c
Sabinene	8.89 ± 0.95 a	5.10 ± 0.21 b	5.22 ± 0.59 b
Myrcene	6.19 ± 0.58 a	6.91 ± 1.08 a	0.53 ± 0.05 b
β-Caryophyllene	1.57 ± 0.74 b	1.20 ± 0.45 b	12.85 ± 0.96 a
(−)-β-Pinene	1.12 ± 0.13 a	0.59 ± 0.05 b	1.23 ± 0.10 a
γ-Terpinene	0.65 ± 0.08 a	0.41 ± 0.05 b	0.50 ± 0.05 b
α-Humulene	—	0.75 ± 0.24 b	1.56 ± 0.04 a
Δ-Cadinene	—	—	4.81 ± 0.23 a
Cineole	27.48 ± 1.01 a	21.02 ± 1.19 b	——
Germacrene D	—	1.62 ± 0.37 a	——
Tricyclo[4.4.0.02,7]decane,1-methyl-3-methylene-8-(1-methylethyl)-, (1R,2S,6S,7S,8S)-rel-	——	——	15.49 ± 2.14 a
(+)-Dipentene	5.25 ± 0.67 b	8.97 ± 1.18 a	——
(1R)-(+)-α-Pinene	4.90 ± 0.76 a	3.99 ± 0.51 a	——
Terpinyl acetate	2.99 ± 0.17 b	5.66 ± 0.40 a	——
Linalyl acetate	7.68 ± 0.77 a	——	——
(−)-α-Pinene	——	—	6.40 ± 0.16 a
(Z)-3,7-Dimethyl-1,3,6-octatriene	——	——	5.59 ± 0.20 a
(+)-α-Pinene	4.90 ± 0.76 a	——	——
γ-Elemene	——	——	4.73 ± 0.23 a
Bicyclo[8.1.0]undeca-2,6-diene	——	——	3.36 ± 0.31 a
Ocimene	2.12 ± 0.21 a	0.90 ± 0.19 b	——
(E)-4-Hexen-1-ol	——	——	2.87 ± 0.70 a
1-Hexanol	0.69 ± 0.34 b	——	1.82 ± 0.69 a
α-Terpineol	1.78 ± 0.08 a	—	——
(−)-Terpinen-4-ol	1.24 ± 0.08 a	0.87 ± 0.09 b	——
cis-1-methyl-4-(propen-2-yl)cyclohexan-1-ol	0.88 ± 0.04 a	0.51 ± 0.07 b	——
Naphthalene,1,2,3,4,4a,5-hexahydro-4,7-dimethyl-1-(1-methylethyl)-, (1s,4s,4as)-	——	——	1.51 ± 0.76 a
α-Muurolene	——	—	1.50 ± 0.04 a
Naphthalene,1,2,3,4-tetrahydro-1,6-dimethyl-4-(1-methylethyl)-, (1r,4r)-rel-	——	——	1.33 ± 0.13 a
β-Elemene	—	——	0.72 ± 0.23 a
Trans-3-hexen-1-ol	1.05 ± 0.75 a	——	——
Trans-2-hexenal	——	——	1.03 ± 0.24 a
(−)-α-Cubebene	——	——	0.75 ± 0.02 a
(+)-Aromadendrene	——	——	0.75 ± 0.02 a
(−)-Limonene	——	——	0.74 ± 0.05 a
Bicyclo[3.1.0]hex-2-ene	0.53 ± 0.04 a	—	——
Naphthalene,1,2,3,4,4a,5,6,7-octahydro-4-methyl-7-methylene-1-(1-methylethyl)-, (1S,4S,4aR)-	——	——	0.70 ± 0.04 a
Naphthalene,1,2,4a,5,6,8a-hexahydro-4,7-dimethyl-1-(1-methylethyl)-, (1S,4aR,8aR)-	——	——	0.66 ± 0.06 a
Dicyclohexene[3.1.0]2-methyl	——	——	0.51 ± 0.05 a

**Table 3:** Factor analysis of leaf volatiles in different *Z. bungeanum* varieties. Factor 1 and Factor 2 explain 66.53% and 27.87% of the total variance, respectively. The multiple comparison data in the table represent mean ± SE. Different lowercase letters within the same column indicate significant differences in factor scores for volatile components among different varieties (one-way ANOVA and Tukey’s HSD test, *p* < 0.05).

Compound Name	Factor 1 (66.53%)	Factor 2 (27.87%)
Linalool	−0.661	0.722
Sabinene	−0.205	0.956
Myrcene	−0.965	0.173
β-Caryophyllene	0.958	−0.256
(−)-β-Pinene	0.501	−0.075
γ-Terpinene	0.148	0.924
α-Humulene	0.695	−0.699
Δ-Cadinene	0.958	−0.276
Cineole	−0.872	0.486
Germacrene D	−0.703	−0.682
Tricyclo[4.4.0.02,7]decane,1-methyl-3-methylene-8-(1-methylethyl)-, (1R,2S,6S,7S,8S)-rel-	0.947	−0.279
(+)-Dipentene	−0.968	−0.124
(1R)-(+)-α-Pinene	−0.884	0.418
Terpinyl acetate	−0.969	−0.200
Linalyl acetate	−0.244	0.958
(−)-α-Pinene	0.960	−0.277
(Z)-3,7-Dimethyl-1,3,6-octatriene	0.959	−0.277
(+)-α-Pinene	−0.242	0.953
γ-Elemene	0.958	−0.277
Bicyclo[8.1.0]undeca-2,6-diene	0.954	−0.278
Ocimene	−0.622	0.773
(E)-4-Hexen-1-ol	0.941	−0.258
1-Hexanol	0.909	0.121
α-Terpineol	−0.247	0.955
(−)-Terpinen-4-ol	−0.832	0.544
cis-1-methyl-4-(propen-2-yl)cyclohexan-1-ol	−0.754	0.645
Naphthalene,1,2,3,4,4a,5-hexahydro-4,7-dimethyl-1-(1-methylethyl)-, (1s,4s,4as)-	0.857	−0.256
α-Muurolene	0.960	−0.275
Naphthalene,1,2,3,4-tetrahydro-1,6-dimethyl-4-(1-methylethyl)-, (1r,4r)-rel-	0.959	−0.270
β-Elemene	0.915	−0.271
Trans-3-hexen-1-ol	−0.181	0.855
Trans-2-hexenal	0.943	−0.259
(−)-α-Cubebene	0.960	−0.276
(+)-Aromadendrene	0.960	−0.276
(−)-Limonene	0.961	−0.275
Bicyclo[3.1.0]hex-2-ene	−0.242	0.968
Naphthalene,1,2,3,4,4a,5,6,7-octahydro-4-methyl-7-methylene-1-(1-methylethyl)-, (1S,4S,4aR)-	0.959	−0.277
Naphthalene,1,2,4a,5,6,8a-hexahydro-4,7-dimethyl-1-(1-methylethyl)-, (1S,4aR,8aR)-	0.960	−0.272
Dicyclohexene[3.1.0]2-methyl	0.960	−0.271
*F*	815.85	138.81
df	2.6	2.6
*p*	<0.001	<0.001
Multiple comparisons		
*Z. bungeanum* Feng	−0.95 ± 0.06 c	−0.91 ± 0.13 b
*Z. bungeanum* Dahongpao	−0.33 ± 0.07 b	1.28 ± 0.25 a
*Z. bungeanum* Fugu	1.28 ± 0.08 a	−0.37 ± 0.08 c

## Data Availability

The original contributions presented in this study are included in the article. Further inquiries can be directed to the corresponding author.
